# Abnormalities of foot and ankle alignment in individuals with chronic ankle instability: a systematic review

**DOI:** 10.1186/s12891-021-04537-6

**Published:** 2021-08-12

**Authors:** Takumi Kobayashi, Yuta Koshino, Takahiro Miki

**Affiliations:** 1 Department of Rehabilitation, Faculty of Health Science, Hokkaido Chitose College of Rehabilitation, 2-10 Satomi, Chitose, Hokkaido, 066-0055 Japan; 2 Rehabilitation Center, NTT Medical Center Sapporo, Sapporo, Hokkaido Japan; 3 Department of Rehabilitation, Sapporo Maruyama Orthopaedic Hospital, Sapporo, Hokkaido Japan

**Keywords:** Ankle instability, Foot posture, Talus, Fibula

## Abstract

**Background:**

To prevent recurrent ankle sprain, it is important to clarify the pathology of chronic ankle instability (CAI). An association has been reported between CAI and abnormalities of foot posture and ankle alignment. There is no consensus on the types of these abnormalities that occur in individuals with CAI. The objective of this systematic review is to clarify the relevance of abnormality of foot posture and ankle alignment for CAI.

**Methods:**

A systematic computerized literature search was performed of the PubMed, CINAHL, SPORTDiscus, Web of Science, and the Cochrane Register of Clinical Trials databases. The selected studies either compared CAI patients with a control group or CAI ankles with contralateral healthy ankles and specifically reported foot posture and alignment of the ankle in the outcomes. They were written in English and published prior to June 2021. The methodological quality of the included studies was evaluated using a 16-question index. Data were extracted independently by two reviewers, and the certainty of evidence was assessed using GRADE approach.

**Results:**

Sixteen studies including 872 patients of high to low methodological quality were included. These showed there was significant anterior displacement and internal rotation of the talus in CAI ankles (low evidence), but there was no consensus on fibular alignment or foot posture.

**Conclusions:**

This review showed there was significant anterior displacement and internal rotation of the talus in CAI ankles but found no consensus on the characteristics of fibular and foot alignment. Further investigations are required to clarify the characteristic foot and ankle malalignment in CAI to facilitate the development of efficient interventions.

**Supplementary Information:**

The online version contains supplementary material available at 10.1186/s12891-021-04537-6.

## Background

Ankle sprain is one of the most common ankle injuries, with a recurrence rate exceeding 50% [1]. Ankle sprains cause mechanical and functional disorders of the ankle joint, and repeated ankle sprains can result in chronic ankle instability (CAI). The main symptoms of CAI are the ankle “giving way”, perceived ankle instability, and further recurrence of ankle sprain [2]. In addition, CAI associated with repeated ankle sprain leads to an increased risk of future ankle osteoarthritis [1]. Recurrent ankle sprains are associated with dysfunction such as limited range of ankle dorsiflexion, decreased external muscle strength, and static and dynamic postural stability deficit [1]. Furthermore, CAI is associated with a decrease in health-related quality of life [3], so clarifying the pathology of CAI is important for developing interventions that can prevent recurrent ankle sprain.

Individuals with CAI experience repeated ankle sprains caused by various dysfunctions, such as pathologic laxity, neuromuscular inhibition, balance deficit, and muscle weakness, including arthrokinematic restrictions caused by abnormal foot and ankle joint alignment such as talar anterior displacement or cavus foot deformity [4]. Abnormal anterior displacement or internal rotation of talus can affect the talar cartilage strain and contribute to the development of osteoarthritis in the future [5, 6]. Abnormalities of foot posture, such as pes planus, have also been noted to potentially alter lower limb kinematics, including increase rearfoot eversion or tibial internal rotation during running, and may thus increase the risk of medial tibial stress syndrome or patellofemoral pain [7]. Abnormalities associated with CAI thus need to be addressed to mitigate long-term consequences.

Rearfoot varus has been reported as a risk factor for CAI [8], and is considered to contribute to biomechanical changes such as increased ankle inversion and lateral deviation of COP during dynamic tasks [9, 10]. In addition, increased navicular drop [11] and posterior deviation of the fibula [12] have been reported as potential risk factors for ankle sprain, although the results have been inconsistent among individuals with CAI [13–16]. Furthermore, the anterior talofibular ligament injury contributes to changes in cartilage loading in the ankle joint by altering talar alignment [5, 6]. For these various abnormalities of foot and ankle alignment, conservative interventions such as joint mobilization or taping to address ankle realignment improved the ankle dorsiflexion range of motion or dynamic postural stability of individuals with CAI [17, 18]. In addition, the surgical repair or reconstruction of the lateral ankle ligament was performed to prevent future osteoarthritis of the ankle [19]. Furthermore, insoles are used for abnormalities of foot posture such as pes planus or cavus foot deformity [20, 21]. However, no consensus has been reached on the types of abnormality of foot posture and ankle alignment that occur with CAI. Although understanding these abnormalities could contribute to the development of more efficient conservative treatments, and ligament repair or reconstruction for CAI, no systematic reviews have clarified the relationship between abnormalities of foot and ankle alignment and CAI. The objective of this systematic review was to clarify the relevance of abnormalities of foot posture and ankle alignment for CAI.

## Methods

### Literature search

This study was conducted and reported according to the PRISMA guidelines for reporting systematic reviews and meta-analysis [22]. A computerized literature search of the PubMed, CINAHL, SPORTDiscus, Web of Science, and the Cochrane Register of Clinical Trials databases was performed on December 6, 2019 and repeated June 15, 2021, and included articles published any time up to that date. The search strategies for each database are shown in Additional file 1: Table A.1-A.4. Articles identified from the search were stored and managed using Endnote X9.

### Inclusion and exclusion criteria

The inclusion criteria were as follows: eligible articles had to be full-text reports of a randomized control trial, cohort study, cross-sectional study, case–control study, or case series published in a peer-reviewed journal; the study compared a group of human participants with CAI with a control group or compared a healthy leg with the contralateral leg with CAI; foot posture and static alignment of the ankle were included in the outcome; and the article was written in English. Because this study included the search period before the publication of recommended criteria by the International Ankle Consortium [2], we also included studies that defined CAI based on a history of ankle sprain, perceived ankle instability, or imaging findings, as well as articles that did not specify the coper subjects. Articles were excluded if the numbers of participants in the CAI and control groups were not clearly presented or quantitative data were not reported for both groups.

### Literature screening

**Fig. 1:**
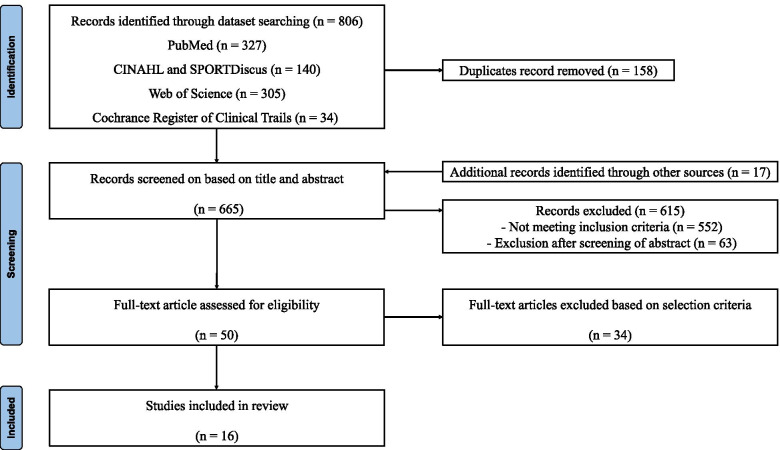
PRISMA flow diagram of the study selection process

After the initial search, articles duplicated between the databases were removed. The titles and abstracts were assessed for relevance to the aim of the review, and the full-text articles were retrieved for those identified as relevant. These were screened against the inclusion and exclusion criteria, and the reference lists of the articles were cross-checked for additional articles. Two reviewers independently screened all the articles identified in the database search, with any disagreements discussed at a meeting between the two reviewers and resolved by consensus. If no consensus was achieved for a specific item, a third reviewer was involved to achieve a consensus between the two raters (Fig. 1).

### Data extraction

All alignment outcome measures, measurements of foot posture and static alignment of the ankle were extracted from the included articles by two independent reviewers. Information about participants, study objectives, definitions of CAI, independent and dependent variables, measurement methods, statistical analyses, main findings, and conclusions were also collected.

### Quality and certainty assessment

Two independent reviewers assessed the risk of bias, with a third reviewer deciding in cases where consensus was lacking. The included studies were evaluated for quality based on an index (Downs and Blacks criteria checklist) developed for nonrandomized studies [23]. Because the present systematic review did not apply questions relating to methodological design validity associated with an intervention, this study used a 16-question version adapted from the original index by Munn et al. (Additional file 1: Table B.1) [24]. The maximum possible score was 17, and the authors recommended that studies scoring < 60% should be considered of low quality, those scoring 60%—74% of moderate quality, and those scoring > 75% of high quality [24]. The independent critical appraisal and data extraction were completed by two reviewers. Any disagreements were resolved by discussion to reach a consensus. Results of the study bias and quality assessment were considered as a component of the outcome certainty assessment. Two independent reviewers assessed the certainty of evidence as “high”, “moderate”, “low”, or “very low” using the Grading of Recommendations Assessment, Development and Evaluation (GRADE) approach [25]. As per the GRADE approach, certainty levels for observational studies started at the “low-certainty” evidence classification level and were upgraded or downgraded according to the set criteria.

**Table 1:**
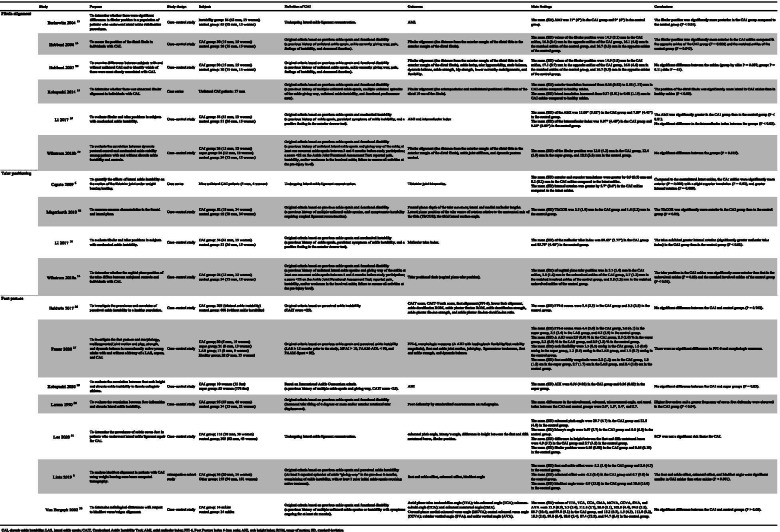
Details of the included studies

## Results

### Study selection and quality

The results of the literature search and review process are presented in Fig. 1. Sixteen papers provided results that were included in the analysis (Table 1) [6, 8, 13–16, 26–35]. The methodological quality scores of the included studies ranged from 6 (35.3%) to 13 (76.5%), with a mean score of 9.69 (57.0%) (Table 2). One study was considered to be of high methodological quality, 5 studies were of moderate quality, and 11 studies were of poor quality.

**Table 2:** Quality assessment of the included studies

Studies (*n* = 16)	Criteria																
	1	2	3	5	6	7	10	11	12	15	16	18	20	21	22	25	Total
Baldwin 2017 [26]	1	1	1	1	1	1	1	0	0	0	1	1	1	1	0	0	11
Berkowitz 2004 [13]	1	1	1	0	1	0	0	0	0	0	1	1	1	1	1	0	9
Caputo 2009 [6]	1	1	1	0	1	0	0	0	0	0	1	0	1	0	0	0	6
Fraser 2020 [27]	1	1	1	1	1	1	0	0	0	0	1	1	1	1	0	0	11
Hubbard 2006 [14]	1	1	1	0	1	0	1	0	0	1	1	1	1	1	0	0	10
Hubbard 2007 [28]	1	1	1	0	1	1	1	0	0	1	1	0	1	1	0	0	10
Kobayashi 2014 [15]	1	1	1	0	1	1	0	0	0	0	1	0	1	0	0	0	7
Kobayashi 2020 [29]	1	1	1	2	1	0	1	0	0	0	1	1	1	1	1	0	12
Larsen 1990 [30]	0	1	0	0	1	1	1	0	0	0	1	0	1	0	0	0	6
Lee 2020 [31]	1	1	1	1	1	1	1	0	0	0	1	0	1	1	1	0	11
Li 2017 [16]	1	1	1	1	1	1	0	0	0	1	1	1	1	0	0	0	10
Lintz 2019 [8]	1	1	1	1	1	1	1	0	0	0	1	1	1	1	1	1	13
Magerkurth 2010 [32]	1	1	1	0	1	1	0	0	0	1	1	1	1	0	0	0	9
Van Bergeyk 2002 [33]	1	1	1	0	1	1	0	0	0	1	1	1	1	0	0	0	9
Wikstrom 2010a [34]	1	1	1	0	1	1	0	0	0	1	1	0	1	1	0	0	9
Wikstrom 2010b [35]	1	1	1	0	1	1	0	0	0	1	1	0	1	1	0	0	12
Mean score																	9.69 (57.0%)

### Fibula alignment

Six studies including 239 patients (39.8 patients per study) investigated fibular alignment [13–16, 28, 35]. Methodological quality was low except for one paper showing moderate quality. Two of these studies recruited patients with CAI who were undergoing lateral ligament reconstruction surgery (only preoperative values were included as results) [13, 16], and the other four studies were based on functional disability, such as recurrent ankle sprains or subjective instability [14, 15, 28, 35]. Two of the papers measured fibular alignment using two-dimensional (2D) analysis in the axial plane based on computed tomography (CT) or magnetic resonance imaging (MRI) images to calculate the axial malleolar index (AMI) [13, 16], three papers used 2D analysis based on sagittal plane radiographs [14, 28, 35], and one study used a three-dimensional (3D) bone model based on CT images [15]. The outcomes measured in these studies were the anterior/posterior and medial/lateral displacement of the fibula.

The two studies involving analysis of the axial plane found the lateral malleolus of the ankle to be significantly posterior in the subjects with CAI [13, 16]. Mean AMI was 11.06–17 degrees in the CAI group and 7.89–9 degrees in the control group [13, 16]. In contrast, of the three analyses using sagittal plane data, one described the lateral malleolus as significantly anterior in the subjects with CAI (mean difference from the control group, 1.8 mm) [14], whereas the others did not detect any significant difference [28, 35]. The 3D analysis did not find a significant difference in anterior/posterior position between the CAI ankles and the contralateral healthy ankles [15]. However, this analysis reported a significant lateral displacement of the fibula (the distal 10 cm of the fibula length) in the CAI ankles compared with the contralateral ankles (Table 1) [15]. The certainty of evidence of fibular anterior/posterior translation could not be determined, because the results were inconsistent. The certainty of evidence for fibular lateral/medial translation was very low (downgraded for bias and imprecision).

### Talar positioning

Four studies including 139 patients (34.8 patients per study) investigated alignment of the talus in CAI [6, 16, 32, 34]. The methodological quality of all papers was low. Two studies recruited patients undergoing lateral ligament reconstruction surgery (only preoperative values were included as results) [6, 16], whereas the other two were based on functional disability, such as recurrent ankle sprains or subjective instability [32, 34]. One study measured talus alignment using a 2D analysis in the axial plane based on MRI images [16], two studies used 2D analysis based on sagittal plane radiographs [32, 34], and one study used a 3D-to-2D registration technique [6]. The measured outcomes were anterior/posterior translation and internal/external rotation of the talus.

The four studies reported consistent results. Regardless of the measurement method and whether or not the ankle was weight bearing, the talus of CAI ankles showed significant anterior displacement (0.9–1.0 mm) and internal rotation (3.85–5.7 degrees) compared with healthy ankles (Table 1) [6, 16, 32, 34]. The certainty of evidence of these results was low.

### Foot posture

Seven studies including 494 patients (70.6 patients per study) investigated foot posture in CAI [8, 26, 27, 29–31, 33]. The methodological quality of one paper was high, with the remaining six showing moderate or low quality. One study recruited patients with CAI who were undergoing lateral ligament reconstruction surgery (only preoperative values were included as results) [31], three of these recruited CAI subjects based on functional disability such as recurrent ankle sprains or subjective instability [8, 30, 33], and the others selected subjects according to scores with the Cumberland Ankle Instability Tool [26, 29] or Identification of Functional Ankle Instability and Foot and Ankle Ability Measure [27]. Two studies measured foot posture using 2D analysis in the frontal/axial plane based on CT images [8, 33], and the other two studies used a 2D analysis of sagittal plane radiographs [30, 31]. In addition, two studies measured from the body surface, applying the Foot Posture Index (FPI-6), [26, 27] and the other two studies calculated arch height index (AHI) or foot mobility magnitude used instrumental measurements (e.g., from a 3D foot scanner) [27, 29]. The measured outcomes were midfoot/rearfoot alignment, including the foot arch height.

The study that used a 2D analysis of sagittal plane radiographs observed mean differences in talocalcaneal, calcaneal, and talometatarsal angles, and tarsal index between the CAI and control groups were 2.0°, 1.5°, 5.4°, and 5.7, respectively [30]. These results suggested a greater frequency of cavus foot deformity in the CAI group than in the control group. In addition, the study using CT images showed mean differences in foot and ankle offset, calcaneal offset, and hindfoot angle between the CAI and control groups of 4.8%, 9.9 mm, and 16.5°, respectively [8]. The authors concluded that hindfoot of CAI ankles was more varus than control ankles. However, the other five studies with image analyses and measurements from the body surface found no significant differences in foot posture between CAI and normal ankles (Table 1) [26, 27, 29, 31, 33]. The certainty of evidence for foot posture could not be determined, because the results were inconsistent.

## Discussion

The aim of this systematic review was to clarify the relationships between CAI and abnormalities of foot posture and ankle alignment. The review included 16 studies that examined fibular or talus alignment or foot posture, retrieved through electronic screening. These indicated significant anterior displacement and internal rotation in the talus of CAI ankles compared with healthy ankles. However, there was no consensus regarding fibular alignment and foot posture.

The results for fibular anterior/posterior translation in CAI ankles differed across the six included studies. Differences in the reference bone may have been the main cause of this variation. Some studies referenced to the tibia and others to the talus, making it difficult to compare findings across the studies. The 3D analysis found the fibulas of CAI ankles were in a significantly lateral position compared with those of the contralateral side [15]. The authors' explanation for this was that lateral displacement of the fibula widened the distal tibiofibular joint, affecting the mortise structure and that this was associated with excessive talar rotation [15]. Fibular repositioning tape is used to treat CAI; this has been shown to improve postural stability [36, 37]. The aim of this approach is to correct fibula alignment using a nonrigid and rigid tape applied from the distal aspect of the fibula, wrapped around the posterior aspect of the leg to apply superoposterior mobilization force to the distal fibula, equal to a Grade IV joint mobilization [36, 37]. However, the findings of the present review showed that the fibulas of CAI ankles may not necessarily be displaced anteriorly, so other interventions, including different taping methods, should also be investigated. Meanwhile, the anterior tibiofibular ligament injury may be involved in some patients with lateral ankle sprains, and this ligament dysfunction may contribute to abnormalities of fibular alignment [15]. In addition, the anterior talofibular ligament and the calcaneofibular ligament was connected at the anterior tip of the lateral malleolus, [38, 39] and repair or reconstruction of these ligaments for CAI may contribute to the improvement of fibular alignment. However, the indications for surgery in patients with CAI are determined based on pain and instability, and the degree of fibular deviation to use as a criterion for surgery remains unclear [40]. However, reliable 3D imaging evaluation is important because abnormal alignment is likely to be associated with functional impairment. In addition, we believe that the tibia should be the criterion when evaluating fibular alignment because the results of this review suggest that CAI causes abnormal anterior displacement and internal rotation in the talus.

The studies included in this systematic review consistently showed significant anterior displacement and internal rotation of the talus of CAI ankles. The main cause for this is thought to be damage to a lateral ankle ligament (such as the anterior talofibular ligament) caused by repeated lateral ankle sprains [6]. These abnormalities can cause limited range of motion, pain, and cartilage damage, which may be associated with the development of osteoarthritis in the future [4, 41]. Wainright et al.[19] have reported that surgical repair of the anterior talofibular ligament in CAI decreased anterior translation and internal rotation of the talus, therefore, surgical repair of the lateral ankle ligament appears to be effective in improving talar alignment. In addition, joint mobilization to improve talar posterior gliding is performed as a treatment for CAI, and has been shown to improve the range of ankle dorsiflexion, ankle plantar-flexion angle at initial contact during a single-leg drop landing, and dynamic postural stability as assessed by posteromedial distance in the star excursion balance test [42, 43]. It is thought that increasing talar posterior gliding improves the anterior displacement of the talus in CAI ankles [44]. In contrast, no effective conservative treatment such as taping or semi-rigid brace for increasing internal rotation of the talus has been reported; this needs further study. Furthermore, no conclusions have been reached regarding the most effective technique for improving talar alignment for preventing osteoarthritis [5, 45].

There was no consensus among the seven studies in this review regarding the characteristics of foot posture in individuals with CAI. In the study by Larsen et al. [30], the foot arches of the CAI group were higher than those of the healthy group, and Lintz et al.[8] concluded that CAI ankles was more varus than control ankles. These two studies that used 2D image analysis indicated abnormalities in CAI, but no foot malalignment in CAI was observed in body surface evaluation studies such as AHI, FPI-6, and foot mobility magnitude [26, 27, 29]. This difference may depend on the measurement methods and the criteria of CAI subjects. Although some studies have examined the relationship between foot alignment and ankle sprain, they came to no conclusion [46]. Further studies are needed on the relevance of foot posture abnormalities for CAI using more reliable instrumented measures or 3D image analysis.

In CAI, abnormal alignment of the talus may contribute to abnormal arthrokinematics and cartilage loading pattern of the talocrural joint, so assessment of talar alignment using reliable modality is useful in clinical practice. As no consensus has been reached regarding the abnormalities of fibular alignment in CAI, intervention methods must be considered according to the specific alignment abnormality in each case. The tibia may also represent the optimal reference for reliable assessment of fibular alignment. In addition, no clear relationship was apparent between foot posture and CAI from the findings of this systematic review. However, since abnormalities of foot posture are suspected to be associated with various clinical dysfunctions, accumulation of further results using reliable methods is important.

This systematic review included 16 retrospective cohort, case–control or cross-sectional studies. Although these provided results regarding the relationship between several aspects of foot or ankle alignment and CAI, the quality of almost included studies was not high, so the evidence underpinning the findings of this systematic review is not strong. In addition, differences in CAI selection criteria among the studies may have influenced the results. For example, some studies included patients with mechanical instability of the ankle joint who resisted conservative treatment, while other studies used a history of ankle sprain and perceived instability as inclusion criteria and did not include the presence or absence of mechanical instability. In addition to mechanical impairments (e.g., arthrokinematics restrictions) and perceptual impairments (e.g., perceived instability), neurophysiological problems were also associated with the pathogenesis of CAI [4]. Future studies should therefore refer to the selection criteria of the International Ankle Consortium to consider coper’s subjects as well [2]. Also, because it is likely that ligamentous dysfunction would affect alignment of the foot and ankle, the presence or absence of mechanical instability should be clarified for each subject. Although foot posture is considered to be influenced by biological sex [47], the present study was unable to consider the effects of sex. Finally, the differences in measurement methods among the studies made it difficult to integrate the results through a meta-analysis. The development of a simple, high-precision measurement method for physicians and therapists is needed.

## Conclusion

The aim of this systematic review was to clarify the relevance for CAI of foot and ankle alignment. The review identified that there was significant anterior displacement and internal rotation in the talus of CAI ankles compared with healthy ankles, but there was no consensus on the characteristics of fibular and foot alignment. In addition, the quality of the studies that investigated foot and ankle alignment was not high. Future studies are needed to clarify the foot and ankle malalignments characteristic of CAI, as well as related ligament injuries, using unified selection criteria. This should facilitate the development of efficient interventions.

## Supplementary Information

**Additional file 1.**

## Data Availability

The datasets used and/or analysed during the current study are available from the corresponding author on reasonable request.

## Abbreviations

CAI: Chronic ankle instability
2D: Two-dimensional
3D: Three-dimensional
CT: Computed tomography
MRI: Magnetic resonance imaging
FPI: Foot Posture Index
AHI: Arch height index
GRADE: Grading of Recommendations Assessment, Development and Evaluation
