# Supplementary material for: Abnormalities of foot and ankle alignment in individuals with chronic ankle instability: a systematic review
Source: BMC Musculoskelet Disord. 2021 Aug 12;22:683. doi: 10.1186/s12891-021-04537-6 (PMC8361650; doi:10.1186/s12891-021-04537-6)
Supplement: Supplementary file 1 — Additional file 1. [file 12891_2021_4537_MOESM1_ESM.docx]

**Table A.1**

**PubMed**

| ＃ | Search term | Number of manuscripts |
| --- | --- | --- |
| 1 | "Ankle"[TW] | 70576 |
| 2 | "Joint Instability"[mh] | 21259 |
| 3 | "Sprains and Strains"[mh] | 19380 |
| 4 | recurrent[tiab] | 299691 |
| 5 | #2 OR #3 OR #4 | 337209 |
| 6 | #1 AND #5 | 5622 |
| 7 | ankle instabilit*[tiab] | 1988 |
| 8 | #6 OR #7 | 6173 |
| 9 | "Leg"[mh] OR "Foot"[mh] | 106346 |
| 10 | "Foot Joints"[mh] | 23713 |
| 11 | "Tibia"[mh] OR "Fibula"[mh] OR "Foot Bones"[mh] | 59619 |
| 12 | tibiofibula*[tiab] | 1963 |
| 13 | #9 OR #10 OR #11 OR #12 | 177014 |
| 14 | "Foot Deformities"[mh] OR "Bone Malalignment"[mh] | 18747 |
| 15 | "Posture"[mh] | 75685 |
| 16 | alignment[tiab] | 80753 |
| 17 | "bone structure"[tiab] | 4129 |
| 18 | "foot arch"[tiab] | 245 |
| 19 | "foot posture"[tiab] | 501 |
| 20 | "arch height"[tiab] | 375 |
| 21 | #14 OR #15 OR #16 OR #17 OR #18 OR #19 OR #20 | 177134 |
| 22 | #13 AND #21 | 16679 |
| 23 | #8 AND #22 | 327 |

**Table A.2**

**CINAHL & SPORTDiscus (within EBSCOhost)**

| ＃ | Search term | Number of manuscripts |
| --- | --- | --- |
| S1 | TX Ankle | 60099 |
| S2 | MH "Joint Instability+" | 10235 |
| S3 | MH "Sprains and Strains+" | 10809 |
| S4 | TX Recurrent | 55833 |
| S5 | S2 OR S3 OR S4 | 74924 |
| S6 | S1 AND S5 | 4547 |
| S7 | TX "ankle instabilit*" | 2857 |
| S8 | S6 OR S7 | 4814 |
| S9 | TX Leg OR TX Ankle OR TX Heel OR TX Metatars* OR TX Toe* OR TX Hallux | 203716 |
| S10 | TX Tibia OR TX Fibula OR TX Talus OR TX Calcaneus | 19386 |
| S11 | TX "Ankle Joint" OR TX talocrural OR TX "Tarsal Joint" OR TX subtalar OR TX tibiofibula* | 10037 |
| S12 | S9 OR S10 OR S11 | 111729 |
| S13 | TX alignment | 23260 |
| S14 | TX malalignment | 2721 |
| S15 | TX "bone structure" | 1044 |
| S16 | TX "foot arch" | 382 |
| S17 | TX "arch height" | 383 |
| S18 | TX "foot posture" | 520 |
| S19 | S13 OR S14 OR S15 OR S16 OR S17 OR S18 | 15535 |
| S20 | S12 AND S19 | 3724 |
| S21 | S8 AND S20 | 140 |

**Table A.3**

**Web of Science**

| ＃ | Search term | Number of manuscripts |
| --- | --- | --- |
| 1 | TS=Ankle Instabilit* OR TS=Recurrent Ankle Sprain* | 4493 |
| 2 | TS=Leg OR TS=Ankle OR TS=Heel OR TS=Metatars* OR TS=Toe* OR TS=Hallux | 232647 |
| 3 | TS=Tibia OR TS=Fibula OR TS=Talus OR TS=Calcaneus | 43001 |
| 4 | TS=Ankle Joint OR TS=talocrural OR TS=Tarsal Joint OR TS=subtalar OR TS=tibiofibula* | 22678 |
| 5 | #2 OR #3 OR #4 | 266566 |
| 6 | TS=alignment OR TS=malalignment OR TS=bone structure OR TS=foot arch OR TS=arch height OR TS=foot posture | 249012 |
| 7 | #5 AND #6 | 10889 |
| 8 | #1 AND #7 | 305 |

**Table A.4**

**Cochrane Library**

| ＃ | Search term | Number of manuscripts |
| --- | --- | --- |
| 1 | Ankle | 10899 |
| 2 | [mh "Joint Instability"] | 760 |
| 3 | [mh "Sprains and Strains"] | 1325 |
| 4 | recurrent | 35861 |
| 5 | #2 OR #3 OR #4 | 37779 |
| 6 | #1 AND #5 | 769 |
| 7 | ankle instabilit* | 658 |
| 8 | #6 OR #7 | 1130 |
| 9 | [mh Leg] OR [mh Foot] | 4141 |
| 10 | [mh "Foot Joints"] | 868 |
| 11 | [mh Tibia] OR [mh Fibula] OR [mh "Foot Bones"] | 906 |
| 12 | tibiofibula* | 106 |
| 13 | #9 OR #10 OR #11 OR #12 | 5746 |
| 14 | [mh "Foot Deformities"] OR [mh "Bone Malalignment"] | 532 |
| 15 | [mh Posture] | 4301 |
| 16 | alignment | 2786 |
| 17 | bone structure | 1427 |
| 18 | foot arch | 286 |
| 19 | foot posture | 674 |
| 20 | arch height | 239 |
| 21 | #14 OR #15 OR #16 OR #17 OR #18 OR #19 OR #20 | 9674 |
| 22 | #13 AND #21 | 610 |
| 23 | #8 AND #22 | 34 |

**Table B.1**

| No. | Classification | Criteria | Score |
| --- | --- | --- | --- |
| 1 | Patients/Outcomes | Is the hypothesis / aim / objective of the study clearly described? | 1 |
| 2 |  | Are the main outcomes to be measured clearly described in the Introduction or Methods section? | 1 |
| 3 |  | Are the characteristics of the subjects included in the study clearly described? | 1 |
| 5 |  | Are the distributions of principal confounders in each group of subjects to be compared clearly described? | 2 |
| 6 | Reported findings/  Statistical analysis | Are the main findings of the study clearly described? | 1 |
| 7 |  | Does the study provide estimates of the random variability in the data for the main outcomes? | 1 |
| 10 |  | Have actual probability values been reported (e.g. 0.035 rather than <0.05) for the main outcomes except where the probability value is less than 0.001? | 1 |
| 11 | External validity | Were the subjects asked to participate in the study representative of the entire population from which they were recruited? | 1 |
| 12 |  | Were those subjects who were prepared to participate representative of the entire population from which they were recruited? | 1 |
| 15 | Internal validity  - Bias | Was an attempt made to blind those measuring the main outcomes? | 1 |
| 16 |  | If any of the results were based on 'data dredging', was this made clear? | 1 |
| 18 |  | Were the statistical tests used to assess the main outcomes appropriate? | 1 |
| 20 |  | Were the main outcome measures used accurate (valid and reliable)? | 1 |
| 21 | Internal validity  - Confounding | Were the subjects (i.e. controls and those with ankle instability) recruited from the same population? | 1 |
| 22 |  | Were study subjects (controls and those with ankle instability) recruited over the same period of time? | 1 |
| 25 |  | Was there adequate adjustment for confounding in the analyses from which the main findings were drawn? | 1 |
